# Association of visual motor processing and social cognition in schizophrenia

**DOI:** 10.1038/s41537-021-00150-7

**Published:** 2021-04-13

**Authors:** Pin-Yen Lu, Yu-Lien Huang, Pai-Chuan Huang, Yi-Chia Liu, Shyh-Yuh Wei, Wei-Yun Hsu, Kao Chin Chen, Po See Chen, Wen-Chen Wu, Yen Kuang Yang, Huai-Hsuan Tseng

**Affiliations:** 1 Jianan Psychiatric Center, Ministry of Health and Welfare, Tainan, Taiwan; 2 Department of Psychology, Fo Guang University, Yilan, Taiwan; 3 Department of Psychiatry, National Cheng Kung University Hospital, College of Medicine, National Cheng Kung University, Tainan, Taiwan; 4 Department of Occupational Therapy, College of Medicine, National Cheng Kung University, Tainan, Taiwan; 5 Institute of Behavioral Medicine, College of Medicine, National Cheng Kung University, Tainan, Taiwan; 6 Department of Psychiatry, National Cheng Kung University Hospital Dou-Liou Branch, Yunlin, Taiwan; 7 School of Medicine and School of Law, Fu Jen Catholic University, New Taipei, Taiwan; 8 Department of Psychiatry, Tainan Hospital, Ministry of Health and Welfare, Tainan, Taiwan

**Keywords:** Biomarkers, Schizophrenia

## Abstract

Patients with schizophrenia have difficulties in social cognitive domains including emotion recognition and mentalization, and in sensorimotor processing and learning. The relationship between social cognitive deficits and sensorimotor function in patients with schizophrenia remains largely unexplored. With the hypothesis that impaired visual motor processing may decelerate information processing and subsequently affects various domains of social cognition, we examined the association of nonverbal emotion recognition, mentalization, and visual motor processing in schizophrenia. The study examined mentalization using the verbal subset of the Chinese version of Theory of Mind (CToM) Task, an equivalent task of the Faux Pas Test; emotion recognition using the Diagnostic Analysis of Nonverbal Accuracy 2-Taiwan version (DANVA-2-TW), and visual motor processing using a joystick tracking task controlled for basic motor function in 34 individuals with chronic schizophrenia in the community and 42 healthy controls. Patients with schizophrenia had significantly worse performance than healthy controls in social cognition, including facial, prosodic emotion recognition, and mentalization. Visual motor processing was also significantly worse in patients with schizophrenia. Only in patients with schizophrenia, both emotion recognition (mainly in prosodic modality, happy, and sad emotions) and mentalization were positively associated with their learning capacity of visual motor processing. These findings suggest a prospective role of sensorimotor function in their social cognitive deficits. Despite that the underlying neural mechanism needs further research, our findings may provide a new direction for restoration of social cognitive function in schizophrenia by enhancing visual motor processing ability.

## Introduction

Schizophrenia is a devastating chronic mental illness with relapsing psychosis, negative symptoms, and prominent deteriorative social dysfunction. Several domains of social cognitive deficits have been observed in patients with schizophrenia^1–5^, including mentalization and emotion recognition. Difficulties in social cognitive function could be an additional symptom dimension and potential trait marker in patients with schizophrenia^6^. These difficulties are correlated with clinical symptom severity^7–11^, particularly negative and cognitive symptoms^12,13^, which may in turn contribute to their symptom presentation and poor interpersonal and social function^14,15^.

Mentalization, a higher level function of social cognition, is defined as “the mental process by which an individual implicitly and explicitly interprets the actions of himself or herself and others as meaningful on the basis of intentional mental states such as desires, needs, feelings, thoughts, beliefs, and fantasies”^16^. Affective mentalizing incorporates inferring an emotional state from nonverbal cues, such as facial expressions^17^. Assumed from available verbal and non-verbal information to others, defects of mentalization related to the feature of mental states have been described in individuals with schizophrenia.

Nonverbal emotional information is often delivered simultaneously across sensory modality via prosodic voices and facial expressions, which is a spontaneous perceptual and cognitive process^18^. A task examining emotional stimuli using both faces and voices helps to access the common emotional processing pathways with less influence of modality-specific factors, such as visual and acoustic acuity, or facial recognition ability^12,19^. The social cognitive deficits of recognizing nonverbal emotions in both facial and prosodic stimuli in schizophrenia, representing their difficulties to collect nonverbal cues accurately, have been well documented^20–24^.

Sensorimotor processing and coordination, like perceiving nonverbal cues of interacting people, play a crucial role in the success of social exchanges. Visual and motor deficits in schizophrenia are associated with the primary symptoms of schizophrenia such as affective flattening, apathy, and disorganization^25^, and with illness severity^26^, social functioning^27^, and functional outcome^28^. In addition, motor deficits and poor social cognition both predict worse functional outcomes^29–36^, implying a link between these two elementary neurocognitive domains in daily functions. Furthermore, Basic sensorimotor processing difficulties are likely to be inherent to the disorder rather than being a consequence of long-term exposure to medication, since they are present early in the illness course and in relatives of individuals with schizophrenia^37^. Together with the early appearance of difficulties in the illness course and the link with negative symptoms and social functioning, it is plausible to assume that basic sensory deficits may result in a decrease in the amount of available and accurate information^38^, and subsequently contributed to the nonverbal recognition deficits that are constantly observed in patient with schizophrenia. Despite that the association between sensory deficits and social cognitive impairments in schizophrenia has been reported repeatedly^39,40^, to the best of our knowledge, only one study^41^ attempted to approach the relationship between both facial and prosodic emotion recognition and visual motor ability directly in schizophrenia in a Caucasian population and found that their social cognitive deficits were at least partly explained by reduced processing speed. Therefore, we hypothesized that visual motor processing impairment may decelerate social information processing and transmission, which may be in turn associated with the difficulties of mentalization and social deficits in schizophrenia. We expected worse performance in both social cognition and visual motor processing in patients with schizophrenia, and a positive association between deficits of visual motor processing and social cognition, including emotion recognition and mentalization. In this study, we examined facial and prosodic emotion recognition through The Diagnostic Analysis of Non-verbal Accuracy 2-Taiwan version (DANVA-2-TW), mentalization by the verbal subset of CToM Task, an equivalent task of the Faux Pas Test, and visual motor coordination and learning abilities using a joystick tracking task, and explored how visual motor processing difficulties may be associated with their social cognitive deficits in schizophrenia while controlling the basic motor function examined by Finger Tapping Test (FTT).

## Results

### Demographic and clinical variables

We have recruited 34 patients (15 males, 19 females) with schizophrenia and 42 healthy controls (17 males, 25 females). The demographic data is listed in Table 1. The mean age of schizophrenia was 40.10 ± 7.86 and was a significant difference (*t* = −2.61, *p* = 0.011) with healthy controls, which was 34.02 ± 11.56. The education years was also a significant difference between the two groups (*t* = 5.17, *p* < 0.001). The mean duration of illness of schizophrenia was 15.03 ± 10.44 years. These patients are chronic and clinically stable (BPRS = 20.24 ± 4.57 and CGI = 3.00 ± 0.35).

**Table 1:** Demographic data and clinical variables.

Variable	Normal (*N* = 42)	Schizophrenia (*N* = 34)	Statistic		
	Mean (SD)	Mean (SD)	*t*/*χ*^2^	*p*	Cohen’s *d*
Age	34.02 (11.56)	40.10 (7.86)	−2.61	0.011*	−0.60
Sex(F/M)	25/17	19/15	0.10	0.749	
Education	17.05 (2.46)	13.44 (3.60)	5.17	0.000*	1.19
Age of onset		23.97 (8.34)			
Duration of illness		15.03 (10.44)			
Hospitalization		3.44 (5.15)			
MMSE total		28.71 (1.57)			
Clinical global impression		3.00 (0.35)			
BPRS		20.24 (4.57)			
Chlorpromazine equivalent dose (mg/d)		326 (214.2)			

*p < 0.05.

### Nonverbal emotion recognition

The emotion recognition accuracy of DANVA-2-TW of patient group and healthy group was displayed in Table 2. Compared to healthy volunteers, patients with schizophrenia had significantly worse performance in both facial (*F* = 10.13, *p* = 0.002) and prosodic emotion recognition (*F* = 37.75, *p* < 0.001) and all four categories of subset emotions, controlled for age and education.

**Table 2:** Score of social cognition, visual motor processing, and Finger Tapping Test.

	Normal (*N* = 42)	Schizophrenia (*N* = 34)	Statistic			
	Mean (SD)	Mean (SD)	*t*	*p*	Cohen’s *d*	*p*^a^
*DANVA-2-TW*						
Total	0.71 (0.11)	0.50 (0.14)	7.25	<0.001*	1.67	<0.001*
Facial total	0.65 (0.14)	0.49 (0.15)	4.78	<0.001*	1.10	0.002*
Prosodic total	0.76 (0.12)	0.50 (0.17)	7.99	<0.001*	1.84	<0.001*
Happy	0.83 (0.08)	0.66 (0.18)	5.40	<0.001*	1.25	<0.001*
Sad	0.67 (0.12)	0.45 (0.16)	6.89	<0.001*	1.59	<0.001*
Angry	0.66 (0.12)	0.48 (0.15)	5.56	<0.001*	1.28	<0.001*
Fearful	0.67 (0.16)	0.39 (0.18)	7.23	<0.001*	1.67	<0.001*
Chinese theory of mind-verbal	26.24 (2.66)	24.53 (4.03)	2.22	0.030	0.51	0.137
*Visual motor processing task*						
Correct score of 1st run	14.32 (8.66)	3.39 (5.56)	6.13	<0.001*	1.46	<0.001*
Correct score of 2nd run	17.49 (9.05)	5.00 (6.73)	6.45	<0.001*	1.53	<0.001*
Correct score of 3rd run	17.85 (8.81)	5.48 (7.02)	6.42	<0.001*	1.53	<0.001*
2nd run–1st run	3.17 (7.29)	1.61 (2.92)	1.12	0.266	0.27	0.513
3rd run–1st run	3.54 (8.02)	2.10 (3.70)	0.93	0.357	0.22	0.359
*Finger tapping test*						
Dominant hand	49.34 (6.82)	35.64 (7.92)	7.77	<0.001*	1.86	<0.001*
Non-dominant hand	46.33 (6.79)	34.93 (7.76)	6.55	<0.001*	1.57	<0.001*

*DANVA-2-TW* diagnostic analysis of non-verbal accuracy 2-Taiwan version.

*p < 0.01 (Bonferroni corrected for five comparisons: DANVA-2-TW total, Chinese theory of mind-verbal, visual motor processing task 1st run, visual motor processing task 3rd run–1st run, Finger tapping test in dominant hand).

^a^Controlled for age and education.

### Mentalization

The mentalization ability measured by CToM verbal subset of patient group and healthy group was displayed in Table 2. Compared to healthy volunteers, patients with schizophrenia had significantly worse performance (*t* = 2.22, *p* = 0.03); however, the significance no longer exist after controlling for age and education (*F* = 2.27, *p* = 0.14).

### Associations between mentalization/social cognition and nonverbal emotion recognition

In patients with schizophrenia, the performance of nonverbal emotion recognition ability was significantly associated with mentalization ability measured by the verbal subtest of CToM after controlled age and education (*r* = 0.49, *p* = 0.005), mainly in happy (*r* = 0.47, *p* = 0.006), sad (*r* = 0.52, *p* = 0.002) and angry (*r* = 0.43, *p* = 0.014) emotions, similarly across facial (*r* = 0.44, *p* = 0.012) and prosodic modality (*r* = 0.45, *p* = 0.010). There was no significant association of CToM and either modality of DANVA-2-TW in normal healthy group (*p* > 0.34).

### Visual motor processing task measures and association with Finger Tapping Test

The visual motor processing task of the patient group and healthy group were revealed as Table 2. Compared to healthy volunteers, patients with schizophrenia performed worse in visual motor coordination (*F* = 24.36, *p* < 0.001), controlled for age and education. The visual motor learning capacity was similar between patient with schizophrenia and healthy controls (*F* = 0.85, *p* = 0.36). The first run visual motor task was not associated with visual motor learning capacity (*r* = 0.08, *p* = 0.688) and FTT (*r* = 0.11, *p* = 0.553) significantly in schizophrenia. There was a significant association between the visual motor learning capacity and FTT (*r* = 0.54, *p* = 0.002) in schizophrenia, which correlation did not reveal in the control groups (*r* = 0.02, *p* = 0.921).

### Visual motor processing and emotion recognition ability

In patient with schizophrenia, nonverbal emotion recognition ability was not associated with the visual motor coordination for the first run (*r* = 0.19, *p* = 0.320), and an incremental association was observed in the second (*r* = 0.33, *p* = 0.082), third run (*r* = 0.33, *p* = 0.047), difference between second and first run (*r* = 0.38, *p* = 0.045), difference between third and first run (*r* = 0.40, *p* = 0.032), respectively, mainly in the prosodic modality (*p* = 0.110–0.009) but not in the face modality (*p* = 0.040–0.98), after controlling for age and education. The association between nonverbal emotion recognition and visual motor coordination was not significant in healthy controls (*p* > 0.48).

Using a linear regression model to explore the association of visual motor coordination, visual motor learning capacity, and social cognition while taking basic motor function, measured by FTT in dominant hand, into consideration, we found that only in patient with schizophrenia, better visual motor learning capacity positively predicted emotion recognition (*β* = 0.48; *p* = 0.037), particularly in prosodic modality (*β* = 0.51; *p* = 0.018) and in happy (*β* = 0.48; *p* = 0.036) and sad (*β* = 0.52; *p* = 0.023) emotion recognition as in Table 3. These associations were not significant in healthy control group (*p* > 0.47) (Table 3).

**Table 3:** Association between visual motor processing task and social cognition tasks after controlling for age and education.

Group	Dependent variables	Correct score of 1st run		3rd run–1st run		FTT	
		*β*	*p*	*β*	*p*	*β*	*p*
Normal	CToM	−0.25	0.253	−0.05	0.786	−0.20	0.287
Schizophrenia		−0.14	0.408	0.57	0.005*	−0.47	0.018*
Normal	DANVA total	0.07	0.733	0.10	0.624	0.17	0.360
Schizophrenia		0.17	0.369	0.48	0.037*	−0.17	0.427
Normal	DANVA facial	0.04	0.863	0.09	0.632	0.20	0.281
Schizophrenia		−0.04	0.859	0.34	0.146	−0.04	0.877
Normal	DANVA prosodic	0.09	0.694	0.06	0.761	0.07	0.720
Schizophrenia		0.33	0.075	0.51	0.018*	−0.27	0.198
Normal	DANVA happy	−0.24	0.252	0.09	0.640	0.00	0.990
Schizophrenia		0.10	0.592	0.48	0.036*	−0.35	0.117
Normal	DANVA sad	0.07	0.751	0.14	0.474	0.20	0.287
Schizophrenia		0.09	0.621	0.52	0.023*	−0.14	0.504
Normal	DANVA angry	0.11	0.571	0.10	0.578	0.19	0.255
Schizophrenia		0.27	0.163	0.43	0.061	−0.24	0.278
Normal	DANVA fearful	0.17	0.421	0.03	0.881	0.15	0.409
Schizophrenia		0.15	0.438	0.27	0.245	0.11	0.619

The maximum value of variance inflation factor in normal group is 1.481 and 1.476 in schizophrenia group.

*DANVA-2-TW* diagnostic analysis of non-verbal accuracy 2-Taiwan version, *CToM* Chinese theory of mind-verbal, *FTT* Finger Tapping Test.

*p < 0.05.

### Visual motor processing and mentalization

In patient with schizophrenia, mentalization was not associated with the visual motor coordination (*p* = 0.058–0.988), after controlling for age and education. The association between nonverbal emotion recognition and visual motor coordination was also not significant in healthy controls (*p* > 0.207).

Following the same regression model, we found that visual motor learning capacity also positively predicted verbal subscale of CToM (*β* = 0.5, *p* = 0.005), while basic motor function negatively predicted verbal subscale of CToM (*β* = −0.47, *p* = 0.018) in patient with schizophrenia. These associations were not significant in healthy control group (*p* > 0.25) (Table 3).

## Discussion

In this preliminary study, we have examined two components of social cognition: mentalization and nonverbal emotion recognition, together with visual motor processing in patients with chronic schizophrenia in community, which were all significantly worse while compared with healthy controls. We observed a significant association between nonverbal emotion recognition and mentalization ability in patient with schizophrenia. We also found a positive association between social cognition and visual motor learning, only in the schizophrenia group.

Our study is the first to examine the correlation between emotion recognition across facial and prosodic modalities and visual motor processing in Han Chinese chronic schizophrenia patients. Previous studies in schizophrenia have demonstrated emotional decoding difficulties in visual^11,42^ or prosodic^10^ stimuli. Studies also demonstrated basic sensorimotor processing deficits^25,39,43,44^ mainly in the magnocellular pathway with reduced contrast sensitivity, and further impacted on the dorsal stream and abnormal motion perception. Although separate evidence suggests that both social cognitive dysfunction and sensorimotor deficits have influenced social interaction and prognosis in schizophrenia^29,30,32–35^, there was not much research have investigated the possible connection, except one^41^ disclosed that patients with schizophrenia were slower and less accurate on emotion recognition, which were significantly associated with longer visual motor reaction time. In line with their observation, we also found a significant association between nonverbal emotion recognition and visual motor learning in patients with schizophrenia. These results were consistent with previous studies reporting global motion processing deficits in schizophrenia, which could contribute to the abnormal object recognition and social functioning^38,43,45^.

The underlying neural correlation between visual motor processing and emotion recognition in schizophrenia remains obscure, and there were few possible speculative explanations. First, visual motor processing and learning may modulate emotional processing through the mirror neuron system^38,46–49^, although some unsupportive arguments exist^50^. Second, evidence supports the association between visual motor processing and emotion recognition may be related to the overlapping modulatory function of the dopaminergic system and subthalamic nucleus, with evidence showing diminished accuracy of emotion recognition in Parkinson’s disease patients with movement disturbances^51–54^. However, the emotion recognition ability as one of the outcome under subthalamic nucleus deep brain stimulation treatment in this population was inconsistent, with improved emotion recognition^55^, unchanged ability^51,56^ or worsening condition^57^. These recent reports could not substantiate the hypothesis currently. Hence, neuroimaging studies exploring the neural correlations are warranted in the future.

Surprisingly, we also observed a significant positive association between emotion recognition ability, particularly in the prosodic modality but not facial modality, and the learning effect of visual motor processing in schizophrenia. Decreased activity of superior temporal sulcus and posterior cingulate gyrus is observed in autistic children during a visuomotor learning task^58^. These regions are also critical regions for deficits of integrating multiple domains of social and non-social information in schizophrenia^24^. A speculation is that the visual motor learning deficits in schizophrenia reflects the difficulty to update and adapt multiple sensory inputs in limited time^59^, which contribute to their social cognitive deficits. In addition, while perceiving audiovisual information separately^12,21^ or concurrently^24,60^, auditory information seems to be more influential in schizophrenia and produced more prominent differences compared to healthy controls, which may provide an explanation for the unexpected stronger association between the prosodic subscale of DANVA-2-TW with visual motor learning task, and lack of an association between the two visual tasks in such a small sample size. Nevertheless, to date there is no research simultaneously approaching the visual and auditory processing, and visual motor ability, and social cognition in schizophrenia. Therefore, our result might provide an aspect for understanding social cognitive deficits in schizophrenia, and a possible route for preserving and improving the social cognitive function in schizophrenia via training for visual motor processing ability.

In addition, for Han Chinese chronic schizophrenia patients, this is the first study to report the significant correlation of nonverbal emotion recognition deficits and verbal mentalization in patients with schizophrenia. Associations between deficits in the theory of mind and emotion recognition in schizophrenia are well-documented in the literature^24,61–66^. In accordance with the literature, our results support nonverbal emotional recognition deficits can be fundamental to their deficits in mentalization/theory of mind in patients with schizophrenia^67^. However, we did not observe a significant association between theory of mind and fear emotion, which has been repeatedly reported to be the least accurate in schizophrenia in the literature^12,61^ and in the current study. A possible explanation is the floor effect of low recognition rate for fear in schizophrenia. Similarly, compared to precedent research^62,68–73^, the correlation between emotion recognition and mentalization in the normal control group was not as prominent, which may partly be explained by the ceiling effect and the relatively small sample size.

In this study, even considering the influence of basic motor function measured by FTT, the association of emotion recognition and visual motor processing remained significant. FTT were significantly correlated with visual motor processing task and negatively associated with CToM. The performance of the FTT has been reported to be associated with Facial Emotion Identification Test in schizophrenia^36^, suggesting a common deficit in perceptual and motor function related to dysfunctional cortico–subcortical circuits. The association was not replicated in our results, which may partially be explained by the different emotional recognition tasks we used and different patient characteristics. Nevertheless, our results at least clarified that the association of social cognitive deficits and visual motor processing in schizophrenia cannot only be explained by basic perceptual and motor function deficits.

The generalizability of the findings might be limited due to several factors. First, the measurement of premorbid IQ was absent. To date, there is no convincing measure for premorbid IQ for Han Chinese, which is a general limitation in this population. Although educational level could be a rough approximation of premorbid IQ, the difference of educational years between study groups was often inevitable due to the early functional impairment in schizophrenia. Second, formal hearing and visual acuity tests were not performed prior to the current study. The estimation of the ability to infer others’ emotions could be inaccurate if deficits did exist within these sensory modalities in our sample. Third, the sample size in our study was relatively small that we were not able to demonstrate the significant associations that were expected, for example, the association between mentalization and emotion recognition in the healthy control group. Fourth, corrections for multiple comparisons of the consecutive regression analyses were not applied in this study. Fifth, although the psychometric properties of the visual motor task were considered adequate, the psychometric properties have not been formally reported before. Readers should be cautious when interpreting the results.

The current study compared emotion recognition, mentalization, and visual motor processing in schizophrenia and healthy control groups and explored the relationships between these abilities. Our results suggest that patients with schizophrenia have difficulties in both aspects of social cognition and visual motor processing. Better visual motor learning capacity seems to be associated with better nonverbal emotion recognition ability, most prominent in the prosodic modality in schizophrenia. We also confirmed the association between emotion recognition and mentalization in schizophrenia in Han Chinese. Foremost, the prominent association between visual motor learning capacity and prosodic emotion recognition may imply a common underlying mechanism of both deficits in schizophrenia, and improving visual motor processing might help to ameliorate social cognition ability in schizophrenia. Further study is warranted to approach the underline neural mechanism of the current observed associations, and the potential effect of visual motor processing training on social cognitive ability in schizophrenia.

## Methods

### Participants

We have recruited 34 chronic stable patients with schizophrenia who met the diagnostic criteria of the Diagnostic and Statistical Manual of Mental Disorders, Fifth Edition, from the community rehabilitation center of National Cheng Kung University Hospital from June 2018 to June 2019. Forty-two healthy volunteers from the community were recruited with the screening of The Mini-International Neuropsychiatric Interview (MINI)^74^ to assess and exclude the psychiatric diagnosis and comorbidity by experienced psychiatrists.

The Institutional Review Board for the Protection of Human Subjects at National Cheng Kung University Hospital approved the research protocols. Upon agreeing to participate in the study, each participant from community rehabilitation center of National Cheng Kung University Hospital was invited for a fully explanation of the study and signed written informed consent of study procedures.

### Study design and procedure

Patients with schizophrenia received clinical interviews and assessments for relevant information, including age of disease onset, duration of illness, time of hospitalization, Clinical Global Impression (CGI), Brief Psychiatric Rating Scale (BPRS), and also Mini-Mental State Examination (MMSE) to exclude any individuals with significant cognitive deterioration. All participants underwent tasks for social cognition, including mentalization (the verbal subtest of the CToM) and emotion recognition (The Diagnostic Analysis of Non-verbal Accuracy 2-Taiwan version, DANVA-2-TW). They also received a joystick tracking task for evaluating visual motor processing. One participant of healthy control and three participants of the patient group did not take the visual motor processing task.

### The Mini-International Neuropsychiatric Interview (MINI)^74^

The MINI is a short, reliable, and valid structured diagnostic interview for psychiatric evaluation. The MINI has good validity and reliability and compares well as a diagnostic instrument with the Composite International Diagnostic Interview (CIDI) and the Structured Clinical Interview for DSM disorders (SCID).

### Clinical global impression (CGI)^75^

The CGI rating scales are measures of symptom severity, treatment response, and efficacy of treatment in treatment studies of patients with mental disorders. It is a 7-point scale that requires the clinician to rate two dimensions of severity of the time of assessment relative to the clinician’s experience, and the improvement relative to a baseline state at the beginning of the intervention.

### Brief psychiatric rating scale (BPRS)^76^

The BPRS is one of the most frequently used instruments for evaluating psychopathology in people with schizophrenia. Its psychometric properties in terms of reliability, validity, and sensitivity have been extensively examined^77^. It measures psychiatric symptoms such as depression, anxiety, hallucinations, and unusual behavior. Each symptom is rated 1 (not present) to 7 (extremely severe).

### The diagnostic analysis of non-verbal accuracy 2-Taiwan version (DANVA-2-TW)^19,78^

The meaning of non-verbal cues can be, at least partially, culturally determined^79^. The DANVA-2-TW, a parallel version of the DANVA 2^80^, is a validated, culturally suitable non-verbal measure for Han Chinese^12,15,19^. The computerized DANVA-2-TW comprises 60 facial photographs and 60 voice clips representing specific emotions, including happy, sad, angry, fearful, and neutral stimuli, to form the facial emotion recognition task and the prosodic emotion recognition task. The voice clips are composed of a semantically neutral sentence, “I am going out of the room now but I’ll be back later”, spoken in various prosodies to reflect the designated emotions.

### CToM task^81^

The CToM task is a validated, published instrument that assesses verbal and non-verbal mentalization capacities. The verbal subset of CToM is an equivalent task of the Faux Pas Test^82^ that has been used frequently in other studies. In this task, participants quietly read ten short stories describing social misbehavior without time limits, and they may read the stories repeatedly. The participants then identify and describe the possible feelings experienced by the characters in the story, when the main character made socially inappropriate statements and hurt or insulted other characters. Each story had four questions and the first question is the memory test. Other questions, such as “What is the person thinking or his motivation?” and “What is the person feeling?”, focuses on the cognitive and affective aspect of CToM individually. The first question must be answered correctly to calculate the scores of the other three questions. Each correct response is scored as 1 point, so the maximum scores for the verbal subset of CToM are 30. For the Chinese version, the internal consistency reliability is .91, and the test–retest reliability is 0.89^81^.

### Visual motor processing task

The visual motor processing task has been used in several animal and human studies^83–85^ with related to premotor cortex activation of motor movement and co-activated with middle temporal and superior temporal regions by human tCDS^83^ and fMRI^84^ exam. The basic psychometric properties of the task in human were considered adequate.^84^ The participant is instructed to use the joystick provided to track the round dots randomly showed on the screen and the task was divided into three blocks (12 min each) with 41 trials each, and the initial correct score and the learning curve were both evaluated. Before the formal tasks, we demonstrated the task at first and made all the participants to practice to follow the target dot with the joystick five times to ensure they understand the task instruction. The measure included the three runs with 41 trials of each without the five rounds previous to the formal round. The program was provided by Thomas RECORDING GmbH (Germany). The movement of the joystick was recorded continuously, and a yellow dot measuring 9 mm was displayed to provide real time feedback. Before the start of the task, the subject was asked to move the joystick to the maximal deflection going into each corner of the screen 2–3 times, then into the maximum circular deflection for another two times for calibration. The target dot measuring 12 mm with a tolerance of until 9 mm would appear in the middle of the screen. The target had a constant speed of 35 mm/s and travelled in a 70 mm target path length. They had to stop when the target stopped on the opposite side of the screen. A correct score was obtained if the cursor color remained yellow from the time the subject positioned it in the middle of the screen. Errors occurred when the feedback cursor moved to the target before it reached its position in the middle of the screen, moved away from the target dot while following its movement, or did not stop after the target already stopped. The cursor dot turned red when any of the mention errors were committed. All participants received three rounds of the visual motor processing task. The scores of each run was collected, and the second and third run score minus the first run score was calculated as an index of visual motor learning ability.

### Finger tapping test^86^

The FTT assesses motor control with evaluating the tapping speed of the fingers and the time between each tap. During administration, the participants’ palm stays in immobile with fingers extended and the index finder placed on the counting device. One hand at a time, participants tap their index finger on the lever as quickly as possible within a 10-s time interval to increase the tapping number. The original procedure calls for five consecutive trials within a 5-point range for each hand, but variations include a total of six trials, in two sets of three. Results from FTT reveal motor impairment or lateralized brain dysfunction.

### Statistical analyses

We employed descriptive statistics to examine basic demographic data and clinical data (age, gender, education, illness onset, and duration of illness), and compared the patient population and healthy controls using *t*-test (continuous variables) and chi-square test (categorical variables). We used analysis of covariance (ANCOVA) to compare between-group differences of the main measures: nonverbal emotion recognition, mentalization, and visual motor coordination (1st run) and learning (calculated as the performance of 3rd run–1st run), controlled for age and education. Partial correlations, controlled for age, and education, among nonverbal emotion recognition, mentalization, and visual motor coordination were performed.

Linear regression model in each group, with social cognitive variables as the dependent variables, and visual motor coordination, visual motor learning, and basic motor function (FTT of the dominant hand) as independent variables, in order to explore the relationships between social cognitive function and visual motor function, was examined.

SPSS Statistics 20.0 (SPSS Inc., Chicago, IL) was used for all of the analyses. The group comparison results were considered significant at *p* < 0.01 (two-tailed, Bonferroni corrected for five main comparisons: DANVA-2-TW total, CToM-verbal, visual motor processing task 1st run, visual motor processing task 3rd run-1st run, FTT of dominant hand.) The significant threshold for the consecutive regression analyses and demographic data were considered significant at *p* < 0.05.

### Reporting summary

Further information on research design is available in the Nature Research Reporting Summary linked to this article.

## Supplementary information

Reporting Summary

## Data Availability

The data used to support the findings of this study are available from the corresponding author upon request.
