# Supplementary material for: Association of visual motor processing and social cognition in schizophrenia
Source: NPJ Schizophr. 2021 Apr 13;7:21. doi: 10.1038/s41537-021-00150-7 (PMC8044174; doi:10.1038/s41537-021-00150-7)
Supplement: Supplementary file 1 — Reporting Summary [file 41537_2021_150_MOESM1_ESM.pdf]

nature research

Corresponding author(s): Huai-Hsuan Tseng

Last updated by author(s): Nov 30, 2020

# Reporting Summary

Nature Research wishes to improve the reproducibility of the work that we publish. This form provides structure for consistency and transparency in reporting. For further information on Nature Research policies, see our Editorial Policies and the Editorial Policy Checklist.

## Statistics

For all statistical analyses, confirm that the following items are present in the figure legend, table legend, main text, or Methods section.

| n/a | Confirmed | |
| --- | --- | --- |
| ☐ | ☒ | The exact sample size ($n$) for each experimental group/condition, given as a discrete number and unit of measurement |
| ☐ | ☒ | A statement on whether measurements were taken from distinct samples or whether the same sample was measured repeatedly |
| ☐ | ☒ | The statistical test(s) used AND whether they are one- or two-sided<br>*Only common tests should be described solely by name; describe more complex techniques in the Methods section.* |
| ☐ | ☒ | A description of all covariates tested |
| ☐ | ☒ | A description of any assumptions or corrections, such as tests of normality and adjustment for multiple comparisons |
| ☐ | ☒ | A full description of the statistical parameters including central tendency (e.g. means) or other basic estimates (e.g. regression coefficient) AND variation (e.g. standard deviation) or associated estimates of uncertainty (e.g. confidence intervals) |
| ☐ | ☒ | For null hypothesis testing, the test statistic (e.g. $F$, $t$, $r$) with confidence intervals, effect sizes, degrees of freedom and $P$ value noted<br>*Give P values as exact values whenever suitable.* |
| ☒ | ☐ | For Bayesian analysis, information on the choice of priors and Markov chain Monte Carlo settings |
| ☒ | ☐ | For hierarchical and complex designs, identification of the appropriate level for tests and full reporting of outcomes |
| ☐ | ☒ | Estimates of effect sizes (e.g. Cohen's $d$, Pearson's $r$), indicating how they were calculated |

*Our web collection on statistics for biologists contains articles on many of the points above.*

## Software and code

Policy information about availability of computer code

| | |
| --- | --- |
| Data collection | SPSS Statistics 20.0 (SPSS Inc., Chicago, IL) |
| Data analysis | SPSS Statistics 20.0 (SPSS Inc., Chicago, IL) |

For manuscripts utilizing custom algorithms or software that are central to the research but not yet described in published literature, software must be made available to editors and reviewers. We strongly encourage code deposition in a community repository (e.g. GitHub). See the Nature Research guidelines for submitting code & software for further information.

## Data

Policy information about availability of data

All manuscripts must include a data availability statement. This statement should provide the following information, where applicable:

- Accession codes, unique identifiers, or web links for publicly available datasets
- A list of figures that have associated raw data
- A description of any restrictions on data availability

The data used to support the findings of this study are available from the corresponding author upon request.

# Field-specific reporting

# Behavioural & social sciences study design

All studies must disclose on these points even when the disclosure is negative.

| | |
|---|---|
| Study description | quantitative cross-sectional |
| Research sample | 34 chronic stable patients with schizophrenia, age 40.10 (7.86), Sex(F/M) 19/15<br>Forty-two healthy volunteers from the community, age 34.02(11.56), Sex(F/M) 25/17 |
| Sampling strategy | convenience, no sample-size calculation was performed |
| Data collection | pen and paper, computer. research assistant was present besides the participants and blind to experimental condition. |
| Timing | May 2016~December 2019 |
| Data exclusions | no |
| Non-participation | no |
| Randomization | participants were not allocated into experimental groups |

# Reporting for specific materials, systems and methods

We require information from authors about some types of materials, experimental systems and methods used in many studies. Here, indicate whether each material, system or method listed is relevant to your study. If you are not sure if a list item applies to your research, read the appropriate section before selecting a response.

## Materials & experimental systems

| n/a | Involved in the study | |
|---|---|---|
| ☒ | ☐ | Antibodies |
| ☒ | ☐ | Eukaryotic cell lines |
| ☒ | ☐ | Palaeontology and archaeology |
| ☒ | ☐ | Animals and other organisms |
| ☐ | ☒ | Human research participants |
| ☒ | ☐ | Clinical data |
| ☒ | ☐ | Dual use research of concern |

## Methods

| n/a | Involved in the study | |
|---|---|---|
| ☒ | ☐ | ChIP-seq |
| ☒ | ☐ | Flow cytometry |
| ☒ | ☐ | MRI-based neuroimaging |

## Human research participants

Policy information about studies involving human research participants

| | |
|---|---|
| Population characteristics | see above |
| Recruitment | 34 chronic stable patients with schizophrenia, age 40.10 (7.86), Sex(F/M) 19/15<br>Forty-two healthy volunteers from the community, age 34.02(11.56), Sex(F/M) 25/17<br>no potential bias |
| Ethics oversight | National Cheng Kung University Hospital |

Note that full information on the approval of the study protocol must also be provided in the manuscript.
